# Structural and Biological Studies of Bioactive Silver(I) Complexes with Coumarin Acid Derivatives

**DOI:** 10.3390/molecules29214993

**Published:** 2024-10-22

**Authors:** Anna Wolska, Aleksandra Drzewiecka-Antonik, Cristina Aparecida Barboza, Marta Struga, Joanna Stefanska, Pawel Rejmak, Marcin Klepka

**Affiliations:** 1 Institute of Physics, Polish Academy of Sciences, Aleja Lotnikow 32/46, PL-02668 Warsaw, Poland; adrzew@ifpan.edu.pl (A.D.-A.); crissetubal@ifpan.edu.pl (C.A.B.); mklepka@ifpan.edu.pl (M.K.); 2 Chair and Department of Biochemistry, Medical University of Warsaw, Banacha 1, PL-02097 Warsaw, Poland; marta.struga@wum.edu.pl; 3 Department of Pharmaceutical Microbiology and Bioanalysis, Centre for Preclinical Research, Medical University of Warsaw, Banacha 1B, PL-02097 Warsaw, Poland; joanna.stefanska@wum.edu.pl

**Keywords:** metal-organic ligand complexes, XAFS, ATR-IR spectroscopy, structural analysis, antimicrobial activity

## Abstract

Two new Ag(I) complexes with coumaric carboxylic acid derivatives have been synthesized. Structural studies of these noncrystalline complexes have been performed using a methodology that combines laboratory and synchrotron techniques, supported by density functional theory calculations. The arrangement of ligands around the Ag(I) cation has been refined using infrared, extended X-ray absorption fine structure, and X-ray absorption near edge structure spectroscopies. Different coordination modes of carboxylate ligands are observed for the studied compounds. Carboxylate bridges are characteristic for the Ag(I) complex with 4-oxo-4*H*-1-benzopyran-2-carboxylic acid (**1**), while a bidentate chelating motif was found for the complex with 2-oxo-2*H*-1-benzopyran-3-carboxylic acid (**2**). Additionally, the carbonyl oxygen atom of the coumarin ring coordinates to the silver cation in complex **2**, while it is inactive in complex **1**. Antimicrobial evaluation has been performed for both compounds. The complexes show activity against selected bacteria as well as *Candida* yeast. This activity is slightly lower for bacteria and the same or higher for *Candida* in relation to the reference substances: ciprofloxacin or fluconazole.

## 1. Introduction

Silver and its salts have been used as therapeutics since antiquity. Today, silver compounds, usually Ag(I) complexes with organic ligands, are used or investigated as antibacterial, antifungal, and anticancer drugs [1,2,3,4,5]. Being nonessential and a relatively low toxicity element for mammals, including humans, silver complexes exhibit strong bactericidal properties, even against bacterial strains possessing high resistivity against antibiotics [1,5].

Having a d^10^ valence configuration, Ag(I) shows little stereochemical preference and can adopt a variety of coordination numbers, spanning from 2 to 8, though low coordination numbers (4, 3, and 2) are preferred [6]. The observed Ag(I) coordination often stems rather from crystal packing and interligand interactions than from weak Ag—ligand binding [6]. The relationship between the structure and biological activity of Ag(I) complexes is not well understood. It is commonly assumed that antimicrobial activity is attributed to Ag(I) ions; hence, the role of the ligand relies mainly on increasing the bioavailability of silver. Complexes of Ag(I) ions with O-donor ligands, being a soft Lewis acid and a hard Lewis base, respectively [7], are relatively weakly bound and labile, which makes them potentially convenient Ag(I) carriers for biological applications. Notably, various Ag(I) complexes with carboxylate ligands have been synthesized, showing both promising antitumor and antimicrobial properties, along with a variety of coordination modes [4,5].

Among the studied bioactive Ag(I) complexes with O-donor ligands, those with coumarin derivatives have so far attracted moderate attention despite the fact that coumarin derivatives and their complexes exhibit a wide range of bioactive behaviors [8,9] as also demonstrated by studies performed in our group [10,11,12]. Ag(I) complexes with hydroxynitrocoumarins [13,14], derivatives of coumarin-3-carboxylic acid [15], co-complexes of phenanthroline and coumarin oxyacetates [16], and an ethyl ester of coumarin acid [17] have been synthesized and investigated, revealing promising antimicrobial properties for these compounds.

In the current work, we present our original results on two new Ag(I) complexes with carboxyl derivatives of coumarin, including their synthesis, structural characterization, and antimicrobial tests. Notably, owing to X-ray absorption spectroscopy studies on noncrystalline samples, we were able to propose structural models for these new compounds, which should contribute to the understanding of the pharmacochemistry of Ag(I) complexes.

## 2. Results

In order to resolve the molecular structure of metal–organic ligand complexes in a noncrystalline state, a combination of extended X-ray absorption fine structure (EXAFS) and X-ray absorption near edge structure (XANES) spectroscopies, elemental analysis, attenuated total reflectance infrared (ATR-IR) spectroscopy, and calculations based on density functional theory (DFT) were used. Microbiological activity of the investigated complexes was also performed. These studies included tests against a series of reference strains of bacteria and *Candida* yeasts.

Structural characterization of the complexes was conducted using a methodology developed by the authors for noncrystalline materials [10]. Laboratory methods provide information on the functional groups coordinating to the metal cation and the metal-to-ligand ratio, while EXAFS analysis delivers information about the coordination number and the kind of species surrounding the absorbing cation. Based on that, structural models were proposed and optimized at the DFT level of theory using PBE0/6-31G(d,p). The shape of the XANES spectra is sensitive to the spatial arrangement of the local neighborhood of the absorbing cations. Therefore, calculations of the theoretical XANES spectra were used to select the most probable models.

### 2.1. Synthesis and Structural Characterisation of Ag(I) Complexes

**Figure 1:**
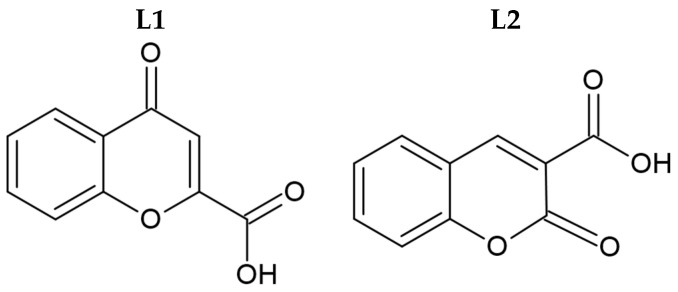
Molecular structure of initial coumarin-based ligands (**L1** and **L2**).

**Figure 2:**
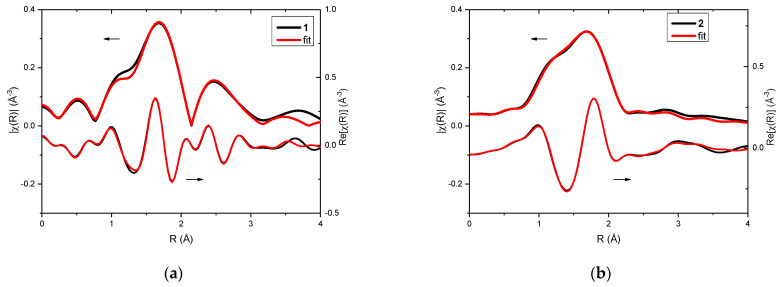
XAFS analysis of complexes **1** (**a**) and **2** (**b**): EXAFS fitting—the magnitudes (upper curves) and real part of Fourier transforms (lower curves) of the experimental oscillations (black line) and the fitting result (red line).

Syntheses of silver(I) complexes with commercially available carboxylic acids, namely 4-oxo-4*H*-1-benzopyran-2-carboxylic acid (**L1**, Figure 1) and 2-oxo-2*H*-1-benzopyran-3-carboxylic acid (**L2**, Figure 1), were conducted in the absence of light. These complexes were synthesized by the deprotonation of the initial ligands with sodium hydride, followed by the addition of equivalent quantities of silver nitrate.

In order to check the purity of the obtained compounds and propose the formulas of the complexes, elemental analysis was performed and mid-infrared spectra were measured and analyzed. Preliminary analysis confirmed the synthesis of two new complexes, **1** and **2**, with **L1** and **L2** ligands, respectively. The complexes turned out to be hydrates, and the ratio of metal cation to organic ligand was 1:1 for both considered compounds.

Infrared spectroscopy allowed an indication of which groups of the coumarin ligands coordinate to the metal cation. Due to deprotonation of the initial acids, two strong bands corresponding to the asymmetric and symmetric stretching motions of the carboxylate group were observed for the complexes. The positions of these bands indicate that complexes **1** and **2** have chelating and/or bridging carboxylate groups [4] since the difference between the values of ν_as_COO^–^ and ν_as_COO^–^ was less than 200 cm^−1^ (being 166 cm^−1^ for **1** and 147 cm^−1^ for **2**). For complexes containing carboxylate ligands coordinating in the monodentate mode, this value, Δν, was significantly above 200 cm^−1^ [4].

Analysis of EXAFS data provided information about the local surrounding around the absorbing silver atoms. In the case of the investigated complexes, **1** and **2**, qualitative comparison already pointed toward differences in their structures (Figure 2a,b). Fitting of EXAFS spectra revealed that in both cases three oxygen atoms can be found in the first coordination sphere (Table 1 and Table 2). For complex **1**, two oxygen atoms were observed at a distance of 2.14 Å from the absorbing Ag(I) and one oxygen atom at a distance of 2.28 Å. However, in the case of complex **2**, one oxygen atom was located closer to the silver cation (Ag…O distance was 2.14 Å), and the remaining two oxygen atoms were slightly further away (Ag…O distance was 2.33 Å).

Moreover, in complex **1**, the Ag atom was located at 2.82 Å and another one was detected at 3.42 Å. In the case of complex **2**, only carbon atoms were found in the further coordination spheres.

In order to propose molecular structures of **1** and **2**, the Cambridge Structural Database (CSD) was searched for structures in agreement with the preliminary EXAFS analysis results, including information from elemental analysis and ATR-IR spectroscopy. Such models, if necessary, were modified and used again for EXAFS spectra fitting. The best models were then used to calculate theoretical XANES spectra, which helped to identify the most probable ones. These results were subsequently employed to build the simplest representative model systems of both complexes with truncated units, as these compounds are polymeric. These models were then used to perform PBE0/6-31G(d,p)/LANL2DZ ground state geometry optimizations.. Both molecules contain coumarins, with the central metal cation bonded to oxygen atoms from the carboxylate moiety. The proposed model for complex **1** suggests the presence of two argentophilic bonds, with bond lengths of 2.82 Å and 3.42 Å. These bonds are crucial in maintaining the offset π-stacking conformation indicated by EXAFS measurements for this compound, which also shows weak coupling between the coumarin units.

In contrast, no experimental evidence of an Ag–Ag bond was found for complex **2**, suggesting that in this complex, the silver(I) cation coordinates to the coumarins through the carboxylate ligand and the keto group at the 2-position of the coumarin. This arrangement places the nearest silver(I) cation over 5 Å from the core, which is beyond the detection range of the EXAFS technique. Furthermore, there is no indication of π-stacking between the coumarin units. The differing coordination modes of these two metallic complexes, as indicated by the EXAFS measurements and supported by the DFT-based calculations, highlight the diversity of silver(I) coordination compounds with coumarin-based ligands. The optimized structures for complexes **1** and **2** were subsequently used in the calculation of XANES spectra.

The final models presenting the molecular geometry of the considered complexes will be presented in the discussion section.

### 2.2. Biological Activity of Ligands and Their Ag(I) Complexes

The obtained complexes (**1** and **2**) were tested for antimicrobial activity against a series of reference strains of bacteria (Gram-positive and Gram-negative) and *Candida* yeasts.

Ciprofloxacin and fluconazole were used as reference substances. It was also investigated whether the activity of the complexes was due to the presence of free silver ions in solution (AgNO_3_). For the tested complexes **1** and **2**, the minimum inhibitory concentration (MIC) values against bacterial strains ranged from 1 to 4 µg/mL. For *Candida* species (*C. albicans* and *C. parapsilosis*), the activity of the complexes was comparable to the antifungal drug fluconazole, with MIC values ranging from 0.5 to 2 µg/mL. Both complexes had significantly higher activity than free silver ions derived from AgNO_3_. The results are presented in Table 3.

In our study, the ligands used in the complexation reactions were inactive against Gram-positive and Gram-negative bacterial strains as well as *Candida albicans*. For ligand **L1**, no results on antimicrobial activity are available in the literature. The unsubstituted ligand **L2** also showed no activity against *Candida albicans*, while derivatives substituted with a bromine atom or a nitrile group were active [18]. Similarly, in our study, ligand **L2** did not show activity against various strains of Gram-positive and Gram-negative bacteria as well as *Candida albicans* [15,19].

## 3. Discussion

**Figure 3:**
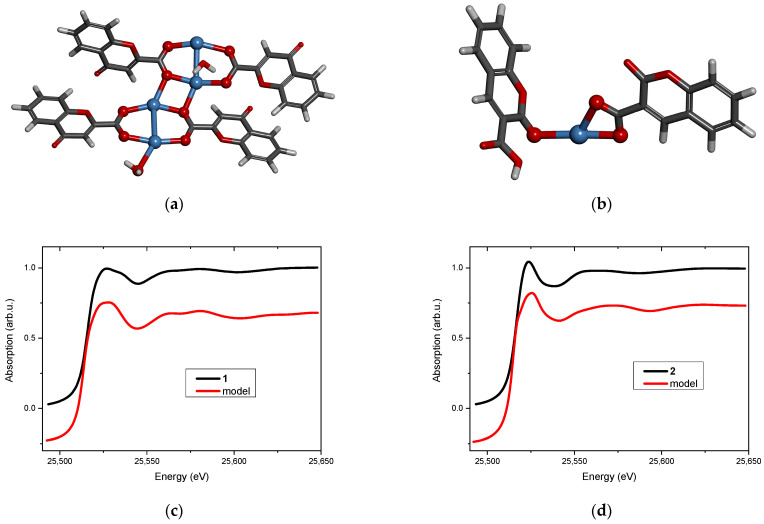
Molecular structure of (**a**) complex **1** and (**b**) complex **2** (silver atoms are marked in blue, oxygen—red, carbon—dark grey, hydrogen—light grey). The XANES spectrum at the Ag K-edge (black line) and the FDMNES simulation based on the model (red line) of (**c**) complex **1** and (**d**) complex **2**.

Two new Ag(I) complexes, labeled **1** and **2**, with 4-oxo-4*H*-1-benzopyran-2-carboxylic acid (**L1**) and with 2-oxo-2*H*-1-benzopyran-3-carboxylic acid (**L2**) were synthesized. Elemental analysis specifies that the new compounds are hydrates and the metal-to-ligand ratio is 1:1. ATR-IR spectroscopy indicates that the complexes contain chelating or/and bridging carboxylate groups. More detailed information was obtained from EXAFS analysis (Table 1 and Table 2), which allowed us to propose molecular structures for the obtained compounds. These models were optimized by DFT calculation, and XANES spectra were calculated for the optimized structures. Then, the theoretical and experimental XANES spectra were compared. The best agreement was obtained for the structures presented in Figure 3a and Figure 3b for complexes **1** and **2**, respectively. Figure 3c,d present the experimental XANES spectra compared with the FDMNES simulations. It can be observed that the main spectral features are reproduced in both cases.

Structural analysis revealed that 4-oxo-4*H*-1-benzopyran-2-carboxylic acid (**L1**) adopts a bridging coordination mode in complex **1** (Figure 3a). Dimeric [Ag_2_L1_2_] units are formed with an Ag…Ag distance of 2.82 Å, which is typical for analogous complexes [20,21]. These dimeric moieties are linked to each other by two short Ag…O interactions (2.3 Å) that ultimately form a polymer. Its tetrameric fragment, used as a model, is shown in Figure 3a.

The coordination sphere of silver(I) in complex **2** is built by two oxygen atoms from the chelating carboxylic group of 2-oxo-2*H*-1-benzopyran-3-carboxylic acid (**L2**) and by one carbonyl oxygen atom from the second ligand molecule (Figure 3b). The ratio of metal to the ligand being 1:1 suggests that the complex has a polymeric structure (most likely a chain) in which the ligand connects silver cations via carboxylate and carbonyl groups.

Complex **1** is a typical representative of carboxylate complexes with bridging COO^–^ groups and short Ag…Ag separation, suggesting the presence of argentophilic interactions [20,21,22]; whereas, complex **2** is a less common example of mononuclear Ag(I) complexes with *O*-donor ligands [23].

Ligands **L1** and **L2,** which were used for synthesis of the complexes, did not show antimicrobial activity. The synthesized complexes were tested for antimicrobial activity against Gram-positive and Gram-negative strains and *Candida* species. The complexes (**1** and **2**) had lower activity against Gram-positive and Gram-negative strains than the reference substance (ciprofloxacin), but the MIC for none of the strains exceeded 4 µg/mL and ranged from 1 to 4 µg/mL. Both complexes had identical activity to the reference substance (fluconazole) against *Candida albicans* ATCC 10231 and ATCC 90028 (MIC 1 or 2 µg/mL). However, against *Candida parapsilosis* ATCC 22019, the activity of the complexes was 4-fold (complex **2**) or 8-fold (complex **1**) higher than that of the reference substance. Compared to silver ions (AgNO_3_), the activity of the complexes was at least four times higher.

## 4. Materials and Methods

### 4.1. Synthesis and Initial Characterisation of the Complexes

The solvents and all chemicals used for the synthesis were commercially available and reagent grade and were applied without further purification. The syntheses of the complexes were conducted in the absence of light, and the final products of the complexation reactions were stored in the dark.

The carbon and hydrogen contents in complexes were determined using a CHN 2400 Perkin–Elmer analyser (PerkinElmer, Waltham, MA, USA). The ATR-IR spectra were recorded over the range of 4000–400 cm^−1^ using a Thermo Scientific Nicolet iS5 spectrometer (Thermo Scientific, Waltham, MA, USA).

The complexes were obtained in two steps. Initially, in situ formed sodium salts of 4-oxo-4*H*-1-benzopyran-2-carboxylic acid (**L1**) and 2-oxo-2*H*-1-benzopyran-3-carboxylic acid (**L2**) were prepared by the addition of 1 mmol NaH (60% pure) to 1 mmol of respective coumarin acids in warm methanol solutions (99% pure, 10 mL). Then, without undue delay, a water solution of AgNO_3_ (1 mmol in 10 mL H_2_O) was added dropwise, resulting in the formation of a white precipitate. A suspension was constantly stirred for 1 h and the resulting precipitate was isolated by filtration, washed with distilled water, and dried at room temperature.

Complex **1**, AgL1·1.5H_2_O, silver(I) complex with 4-oxo-4H-chromene-2-carboxylic acid.

Yield: 70% as a white solid. Anal. Calc. for AgL1·1.5H_2_O: C, 37.07; H, 2.49%. Found: C, 37.23; H, 2.52%. ATR-IR, cm^−1^: 3590, 3489, 3453 (*ν*OH_water_); 3074 (*ν*C-H); 1632 (*ν*C=O_coumarin_); 1595 (*ν*C-C_ring_); 1577 (*ν_as_*COO^–^), 1411 (*ν_s_*COO^–^).

Complex **2**, AgL2·0.5H_2_O, silver(I) complex with 2-oxo-2H-chromene-3-carboxylic acid.

Yield: 75% as a white solid. Anal. Calc. for AgL2·0.25H_2_O: C, 39.25; H, 1.87%. Found: C, 39.05; H, 1.76%. ATR-IR, cm^−1^: 3561, 3405 (*ν*OH_water_); 3050 (*ν*C-H); 1683 (*ν*C=O_coumarin_); 1607 (*ν*C-C_ring_); 1520 (*ν_as_*COO^–^), 1373 (*ν_s_*COO^–^).

### 4.2. X-Ray Absorption Spectroscopy

XAS measurements at the Ag K-edge were performed at the XAFS beamline at the Elettra synchrotron (Trieste, Italy). A double crystal Si 311 monochromator (Kohzu, Kawasaki, Kanagawa, Japan) was calibrated using Ag foil. The powdered complexes were pressed into carbon tape and measured in transmission mode at ambient temperature. During the experiment, no radiation damage was observed. EXAFS data were analyzed using a Demeter package v. 0.9.26 [24]. The k^2^ weighted χ(k) data were Fourier transformed in the k range 2.5–12 Å^−1^ for complex **1** and 3.4–12 Å^−1^ for complex **2**. Theoretical XANES calculations were carried out using a FDMNES code [25,26], applying the ab initio finite difference method. The Hedin-Lundqvist exchange-correlation potential, taking into consideration a core hole effect, was used.

### 4.3. DFT Calculations

Ground-state molecular structures for both models were optimized at the PBE0/6-31G(d,p) level of theory using Gaussian 16 version C.01 [27,28,29,30,31]. This particular density functional was chosen for its balance between computational efficiency and accuracy in predicting the molecular geometries of organometallic complexes. Since calculations were performed for the model systems of both complexes, to avoid artifacts, the first coordination sphere of the central silver(I) cation for both systems was kept constrained during the course of the optimization using bond lengths obtained by EXAFS analysis. In order to incorporate relativistic corrections, which are significant for heavy elements like silver (I), pseudopotential LANL2DZ was used [32,33,34].

### 4.4. Biological Activity Investigations

The antibacterial activity of the compounds was evaluated by the minimal inhibitory concentrations (MIC) method using the serial two-fold dilution method (in 96-well microtiter plates on Mueller-Hinton broth for bacteria and RPMI-1640 medium for *Candida* species) under standard conditions as described in Committee Laboratory Standards (CLSI) and European Committee for Antimicrobial Susceptibility Testing (EUCAST) [32,33]. The final inoculum of bacteria in all studies was approximately 5 × 10^6^ CFU/mL (colony forming unit per mL) and 0.5–2.5 × 10^5^ CFU/mL for yeast.

The studies were conducted using a series of reference strains of bacteria—Gram-positive: *Staphylococcus aureus* (ATCC 25923, ATCC 6538, NCTC 4163), *Staphylococcus epidermidis* (ATCC 35984, ATCC 12228), *Bacillus subtilis* ATCC 6633, *Bacillus cereus* ATCC 11778, *Enterococcus hirae* ATCC 10541, *Enterococcus faecalis* ATCC29212, and *Micrococcus luteus* (ATCC 10240, ATCC 9341); Gram-negative: *Escherichia coli* ATCC 10538, *Proteus vulgaris* NCTC 4635, *Pseudomonas aeruginosa* (ATCC 15442, ATCC 27853), and *Bordetella bronchiseptica* ATCC 4617; and yeasts: *Candida albicans* (ATCC 10231, ATCC 90028) and *Candida parapsilosis* ATCC 22019. The activity of the compounds was determined in a concentration range of 0.125–256 µg/mL. Ciprofloxacin and fluconazole were used as reference compounds.

The MIC for bacteria was defined as the lowest drug concentration that completely inhibited growth of the organism in the microdilution wells as detected by the unaided eye after 18 h of incubation [35].

Yeast growth was evaluated by absorbance measurement at 530 nm after at least 24 h of incubation and until the absorbance of the compound-free well was reached. The MIC was defined as a 50% or more reduction in growth compared to the control well [36].

## 5. Conclusions

Two new Ag(I) complexes with coumarin carboxylic acids have been synthesized. The initial ligands, as well as the complexes, were screened for antibacterial activity against a series of Gram-positive and Gram-negative bacteria and *Candida* yeasts. The complexes had lower activity against bacterial strains than the reference ciprofloxacin with a MIC ranging from 1 to 4 µg/mL. Both complexes had identical activity to the reference fluconazole against *Candida albicans* (MIC equal to 1 or 2 µg/mL), and the activity of the complexes against *Candida parapsilosis* was 4-fold or even 8-fold higher than that of the reference substance.

**Figure 4:**
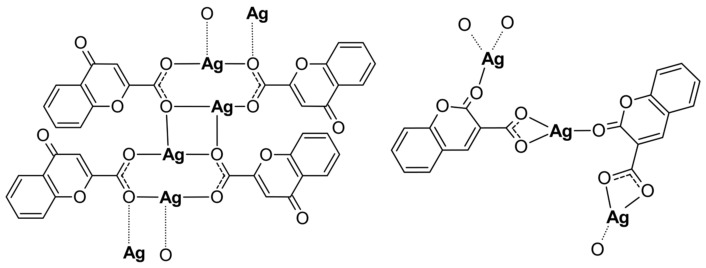
The polymeric structures of analyzed silver complexes (water molecules have been omitted).

Both Ag(I) complexes have been structurally characterized by elemental analysis, ATR-IR, EXAFS, and XANES spectroscopies. The compounds possess a polymeric structure with different carboxylate coordination modes of the initial ligands as presented in Figure 4. Furthermore, the keto O atom, depending on its position in the coumarin system, may or may not enter the coordination sphere of the silver cation. Our investigation revealed that changes in the positions of the carbonyl and acid groups in the organic part of the initial ligands have a significant impact on the structure of their complexes with silver. However, changing the position of the carboxyl group from position 2 to 3 and the carbonyl group from position 4 to 2 in the coumarin system only slightly increased the antimicrobial activity of the obtained complexes.

## Figures and Tables

**Table 1:** The parameters of EXAFS fitting of complex **1**. N—number of atoms in each scattering path, R—distance from the central atom, σ^2^—Debye–Waller factor describing local atomic disorder. The amplitude reduction factor S_0_^2^ was found to be 0.8 (1) and the R-factor of the fit was 0.009.

Atom Type	N	R [Å]	σ^2^ [Å^2^]
O1	2	2.14 (2)	0.002 (1)
O2	1	2.28 (4)	0.002 (1)
Ag1	1	2.82 (8)	0.010 (2)
C1	3	3.06 (7)	0.005 (1)
O3	3	3.27 (7)	0.005 (1)
Ag2	1	3.42 (11)	0.010 (2)

**Table 2:** The parameters of EXAFS fitting of complex **2**. N—number of atoms in each scattering path, R—distance from the central atom, σ^2^—Debye–Waller factor describing local atomic disorder. The amplitude reduction factor S_0_^2^ was found to be 1.0 (1) and the R-factor of the fit was 0.003.

Atom Type	N	R [Å]	σ^2^ [Å^2^]
O1	1	2.14 (3)	0.002 (1)
O2	2	2.33 (3)	0.002 (1)
C1	1	2.54 (4)	0.004 (2)
C2	1	3.04 (6)	0.004 (2)

**Table 3:** Activities of obtained compounds against Gram-positive and Gram-negative bacteria and yeasts—minimal inhibitory concentration (MIC, µg/mL).

Compound/Bacteria and Yeasts Strains	L1	L2	1	2	AgNO_3_	Ciprofloxacin	Fluconazole
*S. aureus* ATCC 25923	>100	>100	2	4	16	0.5	
*S. aureus* NCTC 4163	>100	>100	4	4	16	0.25	
*S. aureus* ATCC 6538	>100	>100	2	2	16	0.5	
*S. epidermidis* ATCC 35984	>100	>100	2	2	32	0.25	
*S. epidermidis* ATCC 12228	>100	>100	2	4	32	0.5	
*B. subtilis* ATCC 6633	>100	>100	2	2	8	≤0.125	
*B. cereus* ATCC 11778	>100	>100	2	2	8	0.25	
*E. hirae* ATCC 10541	>100	>100	2	2	16	2	
*E. faecalis* ATCC 29212	>100	>100	4	4	16	2	
*M. luteus* ATCC 10240	>100	>100	2	2	8	1	
*M. luteus* ATCC 9341	>100	>100	2	1	8	2	
*E. coli* NCTC 10538	>100	>100	2	2	16	≤0.125	
*P. vulgaris* NCTC 4635	>100	>100	2	4	32	≤0.125	
*P. aeruginosa* ATCC 27853	>100	>100	2	2	32	0.5	
*P. aeruginosa* ATCC 15442	>100	>100	2	2	32	0.5	
*B. bronchiseptica* ATCC4617	>100	>100	2	2	32	1	
*C. albicans* ATCC 10231	>100	>100	1	2	16		2
*C. albicans* ATCC 90028	>100	>100	1	1	16		1
*C. parapsilosis* ATCC 22019	>100	>100	1	0.5	16		2

## Data Availability

Dataset available on request from the authors.
